# Small non-coding RNA STnc640 regulates expression of *fimA* fimbrial gene and virulence of *Salmonella enterica* serovar Enteritidis

**DOI:** 10.1186/s12917-019-2066-7

**Published:** 2019-09-05

**Authors:** Xia Meng, Xianchen Meng, Jinqiu Wang, Heng Wang, Chunhong Zhu, Jie Ni, Guoqiang Zhu

**Affiliations:** 1 College of Veterinary Medicine, Yangzhou University, Yangzhou, 225009 China; 2 Jiangsu Co-innovation Center for Prevention and Control of Important Animal Infectious Diseases and Zoonoses, Yangzhou, 225009 China; 3 Department of Animal Husbandry and Veterinary Medicine, Beijing Vocational College of Agriculture, Beijing, 102442 China; 4 Jiangsu provincial key lab for genetics and breeding of poultry, Jiangsu Institute of Poultry Science, Yangzhou, 225125 China

**Keywords:** *Salmonella* Enteritidis, STnc640, Regulation, Adhesion, Virulence

## Abstract

**Background:**

Small non-coding RNAs (sRNAs) regulate bacterial gene expression at the post-transcriptional level. STnc640 is a type of sRNA that was identified in *Salmonella* Typhimurium.

**Results:**

In this study, STnc640 in *Salmonella* Enteritidis was confirmed to be an Hfq-dependent sRNA. TargetRNA software analysis showed that fimbrial genes *fimA* and *bcfA* were likely to be the target genes of STnc640. To investigate the target mRNAs and function of STnc640 in pathogenicity, we constructed the deletion mutant strain 50336△*stnc640* and the complemented strain 50336△*stnc640*/p*stnc640* in *Salmonella* Enteritidis 50336. The RT-qPCR results showed that the mRNA level of *fimA* was decreased, while *bcfA* was unchanged in 50336△*stnc640* compared with that in the wild type (WT) strain. The adhesion ability of 50336△*stnc640* to Caco-2 cells was increased compared to the 50336 WT strain. The virulence of 50336△*stnc640* was enhanced in a one-day-old chicken model of *S.* Enteritidis disease as determined by quantifying the 50% lethal dose (LD_50_) of the bacterial strains.

**Conclusions:**

The results demonstrate that STnc640 contributes to the virulence of *Salmonella* Enteritidis.

## Background

Small non-coding RNAs (sRNAs) in bacteria are stable transcripts approximately 50–500 nucleotides in length, often encoded in intergenic regions (IGRs), that play important roles in regulating gene expression at the post-transcriptional level [1–4]. sRNAs regulate many physiological processes, including metabolism, iron homeostasis, outer membrane protein biosynthesis, quorum sensing, and virulence [5–8]. Many of these sRNAs require the RNA-chaperone Hfq [9]. Nearly 100 distinct sRNAs have been identified in *Salmonella* [10].

*Salmonella enterica* serovar Enteritidis is an important Gram-negative intracellular pathogen with a broad host range. It can infect young chickens and cause symptoms such as enteritis or systemic infection [11]. Adult chickens infected with *Salmonella* Enteritidis may have subclinical infections and become chronic carriers, leading to contamination of chicken meat and egg products and the resulting food-borne diarrheal illnesses in humans [12]. Adhesion to intestinal epithelial cells mediated by bacterial fimbriae is a necessary first step for colonization [13–16]. Whole-genome sequencing has identified 13 fimbriae operons in the *Salmonella* Enteritidis strain P125109 [17]. The *fim* operon directs the assembly of type I fimbriae, which are involved in reproductive tract infection and in egg contamination [15]. Type I fimbriae and other multiple fimbrial adhesins are also required for the colonization of the intestinal lumen and for the virulence of *Salmonella* Typhimurium in mice [18].

STnc640 is a novel Hfq-binding sRNA that was identified in *Salmonella* Typhimurium through deep sequencing and transcriptomic analysis of Hfq-bound sRNAs and mRNAs [19]. Here we constructed a *stnc640* deletion mutant and characterized the role of this sRNA in bacterial adhesion and virulence.

## Results

### Hfq plays a positive role on STnc640 stability

**Fig. 1:**
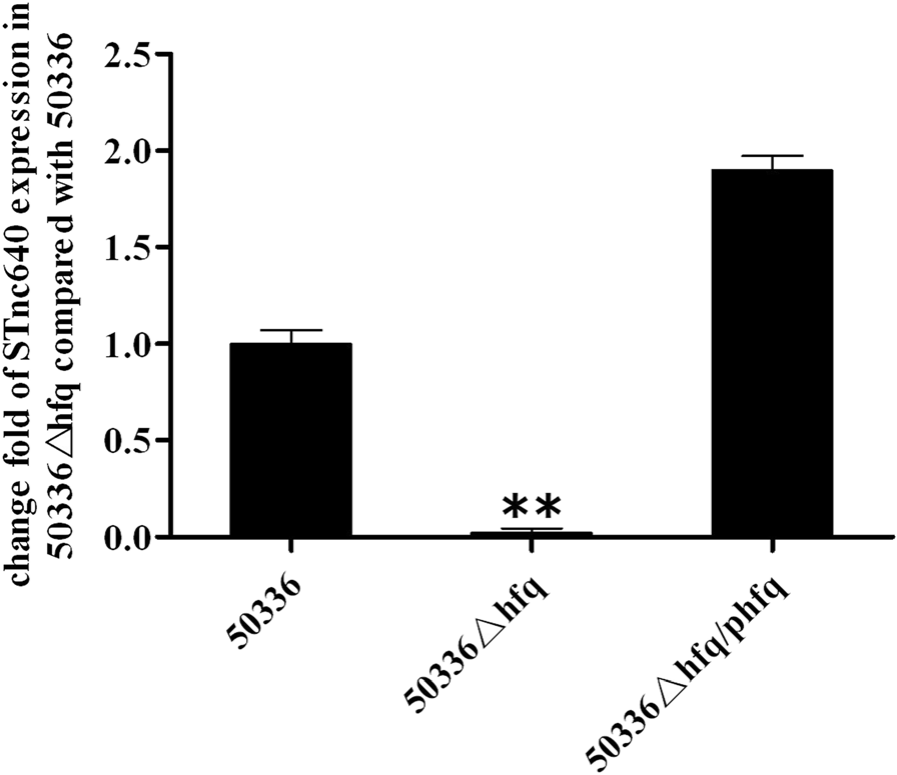
Fold changes of the STnc640 gene mRNA level were measured in the mutant 50336△*hfq* and complementation strain 50336△*hfq*/p*hfq* by RT-qPCR compared with the wild-type *S.* Enteritidis 50336. Assays were performed in triplicate. **Indicates statistically significant difference compared with the wild type strain (*p* < 0.01)

To determine whether the stability of STnc640 depends on the sRNA chaperone protein Hfq, the abundance of *stnc640* transcripts in *S.* Enteritidis WT strain 50336, mutant 50336△*hfq* and the complemented mutant 50336△*hfq*/p*hfq* were determined using RT-qPCR. The abundance of *stnc640* was significantly reduced in 50336△*hfq*, exhibiting only about 2% of that in the WT strain (*P* < 0.01) and was restored in the 50336△*hfq*/p*hfq* mutant (Fig. 1). This indicated that Hfq played a positive role on STnc640 stability.

### Candidate mRNA targets of STnc640

Candidate mRNA targets of STnc640 were predicted using TargetRNA2 [20]. There were nine consecutive hybridization seeds between the AU-rich region of STnc640 (nts 263–277) and *bcfA* (nts 37–51). There were 11 consecutive hybridization seeds between the coding sequences (codons 8–25) of *fimA* mRNA and STnc640 (codons 99–125).

### Construction and growth characteristics of the mutant 50336△stnc640 and complemented strain 50336△stnc640/pstnc640

**Fig. 2:**
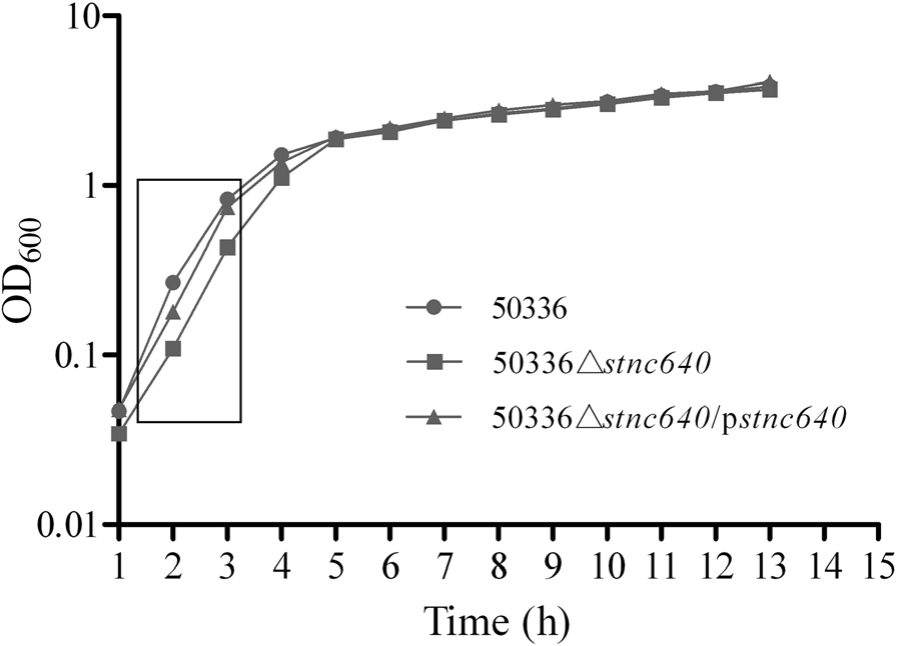
Growth curves of wild-type *S.* Enteritidis 50336, mutant 50336△*stnc640,* and complementation strain 50336△*stnc640*/p*stnc640.* OD_600_ values of triplicate cultures in LB medium were determined at 1 h intervals. Data are the means of three independent experiments. The box in the figure indicates that the growth was significantly reduced from 2 h to 3 h

*S.* Enteritidis strain 50336 contains an *stnc640* gene with 97% identity to the *S.* Typhimurium strain LT2 *stnc640* gene. STnc640 was located in a non-coding region between the genes SEN1810 and *icdA* in S. Enteritidis. In the construction of the deletion and the complemented strains, a 460 bp DNA fragment of the non-coding region was deleted and complemented. We constructed an *stnc640* deletion mutant 50336△*stnc640* and compared its growth to the WT and complemented strains. The growth rate of 50336△*stnc640* was significantly reduced during the log phase from 2 h to 3 h (*P* < 0.05) (Fig. 2).

### STnc640 regulates fimA expression and affects adherence and invasion to Caco-2 cells

**Fig. 3:**
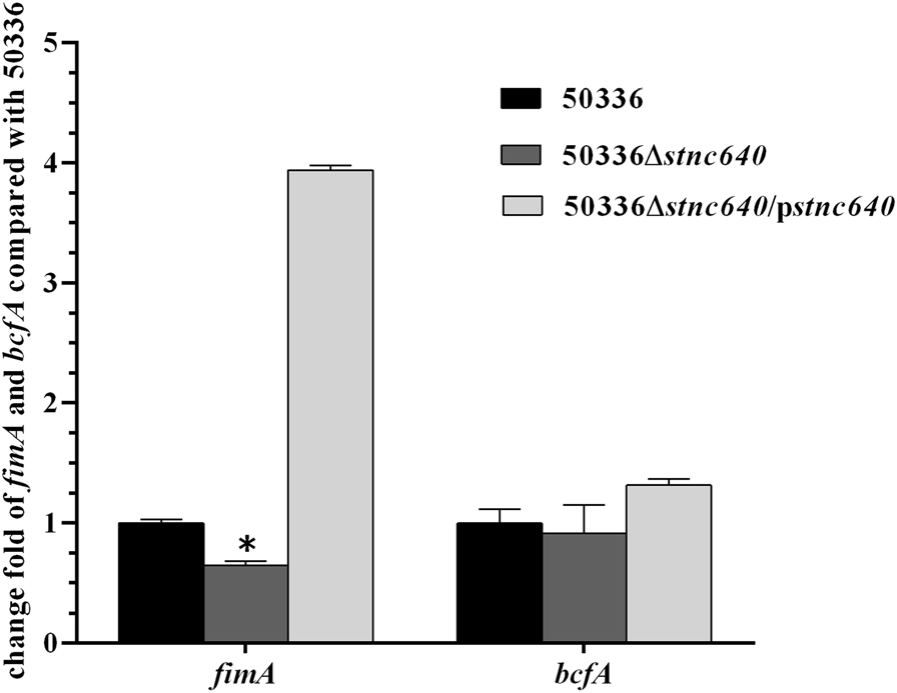
The mRNA levels of fimbrial genes *fimA* and *bcfA* were determined in the mutant 50336△*stnc640* and 50336△*stnc640*/p*stnc640* compared to wild-type *S.* Enteritidis 50336 by qRT-PCR. Assays were performed in triplicate

**Fig. 4:**
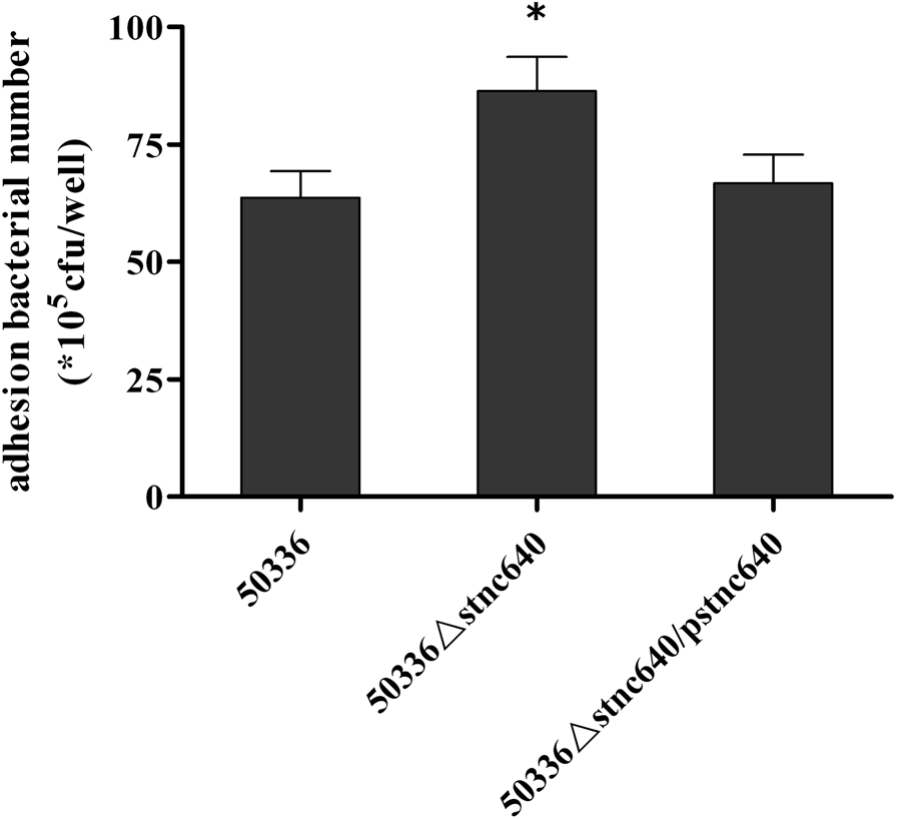
Adherence to Caco-2 cells by wild-type *S.* Enteritidis 50336, mutant 50336△*stnc640,* and complementation strain 50336△*stnc640*/p*stnc640.* Data are expressed as mean ± standard deviation of triplicate experiments. *Indicates statistically significant difference compared with the wild type strain (*p* < 0.05)

To determine whether *bcfA* and/or *fimA* expression are regulated by STnc640, we quantified *bcfA* and *fimA* expression using RT-qPCR. The *fimA* but not *bcfA* transcript abundance was reduced in the Δ*stnc640* mutant compared with the WT strain (Fig. 3). To investigate whether deleting *stnc640* affected bacterial adhesion and invasion by regulating *fimA*, we performed bacterial adhesion and invasion assays. Δ*stnc640* was enhanced in adhering and invading to Caco-2 cells compared with the WT strain (Fig. 4).

### Deleting stnc640 enhances virulence in chickens

LD_50_ assays were performed to analyze the effect of *stnc640* on S. Enteritidis virulence in chickens. All of the chickens displayed intestinal hyperemia and diarrhea 10 h post infection. Higher mortality appeared when infected by 50336△*stnc640* compared to the WT strain and the complemented strain. The mortality rates for 10^7^, 10^8^ and 10^9^ CFU bacteria treatment were 5, 57 and 95% separately when infected by 50336△*stnc640*. The mortality rates for the above three dose treatment were 5, 50 and 85% separately when infected by the WT strain, and the rates were 0, 5 and 62% when infected by the complemented strain. The LD50s were calculated 14 days post-infection. The LD50 values of the WT strain 50336, 50336△*stnc640* and 50336△*stnc640*/p*stnc640* were 2.9 × 10^8^, 2.0 × 10^8^ and 5.1 × 10^8^ CFU, respectively. This indicated that the virulence of 50336△*stnc640* was enhanced approximately 1.5-fold compared with the WT. The virulence of complemented strain 50336△*stnc640*/p*stnc640* has attenuated compared with the WT strain and the 50336△*stnc640* mutant. Tests of isolation and identification of bacteria showed that all three strains of S. Enteritidis were widely distributed in the liver, spleen, and caecum of the infected chickens.

## Discussion

sRNAs are a ubiquitous class of molecules that can regulate gene expression at the post-transcriptional level. Most sRNAs can interact with their target mRNAs by base-pairing actions and then modulate translation, degradation, or stability of mRNA [4]. In this study, an sRNA gene *stnc640* of *S.* Enteritidis strain 50336 was cloned and showed 97% identity with *stnc640* of *S.* Typhimurium. This indicated that *stnc640* has very high homology within the genus. Identification of the STnc640 target gene is important for the study of sRNA function. To date, the target genes and the function of STnc640 remain unknown. We thus identified likely candidate mRNA targets of STnc640 (*fimA* and *bcfA*) by bioinformatics predication technology using TargetRNA2.

The growth rates of the WT strain, 50336△*stnc640,* and 50336△*stnc640*/p*stnc640* were determined by measuring OD_600_. The growth rate of 50336△*stnc640* was lower than those of the WT strain and 50336△*stnc640*/p*stnc640* in the log phase. Many sRNAs can directly sense multiple environmental signals such as fluctuations in temperature, pH, and metabolites [3, 21]. The deletion of STnc640 apparently weakened environmental adaptation, leading to the decline in growth rate at the log phase, but the final concentration of bacteria was not affected.

The STnc640 candidate targets *fimA* and *bcfA* were verified by detecting their mRNA levels by RT-qPCR. The expression of *fimA* was down-regulated in 50336△*stnc640* compared to the WT strain. This suggested that that STnc640 could regulate *fimA* expression. In other words, *fimA* was a likely target of STnc640. However, the regulation mechanism needs further study. *FimA* is a major fimbrial subunit in *Salmonella enterica*. The Type I fimbriae can alter virulence of S. Typhimurium toward mice [18]. Type I fimbriae are also involved in clearance of *S.* Enteritidis from the blood and in egg contamination by *S.* Enteritidis in laying hens [15]. Deletion of STnc640 led to a decrease of fimA expression, but the ability of adhesion to Caco-2 cells of the STnc640 mutant was stronger than that of the wild type strain. This indicated that there is no direct relationship between *fimA* expression and adhesion ability. Rajashekara found that deletion of the *fimA* gene in *S.* Enteritidis did not affect the ability to invade Caco-2 cells and colonize the chicken caecum [22], which is consistent with our result. Multiple fimbrial adhesins are required for *Salmonella* colonization of the chicken intestine tract. We supposed that up-regulation of other adhesion-related genes expression, but not down-regulation of the *fimA* gene, caused the adhesion ability enhancement in the STnc640 deletion mutant. Adhesion to and colonization of host cells are important factors for virulence. In our study, the STnc640 deletion in *S.* Enteritidis strengthened the ability to adhere to Caco-2 cells and thus increased the virulence in chickens. We inferred that STnc640 could inhibit *S.* Enteritidis virulence by affecting adhesion. For further confirm of whether STnc640 could inhibit virulence, overexpression of STnc640 in the wild type strain and comparison that with wild type need to be performed in the future.

## Conclusions

Small non-coding RNA STnc640 could regulate the expression of *fimA* fimbrial gene in *S.* Enteritidis. The deletion of STnc640 in *S.* Enteritidis strengthened the ability to adhere to and colonize in Caco-2 cells and thus increased the virulence in chickens. It was supposed that STnc640 could inhibit *S.* Enteritidis virulence by affecting adhesion.

## Methods

### Bacterial strains, plasmids and cell culture conditions

The bacteria strains and plasmids used in this study are listed in Table 1. *Salmonella* Enteritidis wild type (WT) strain 50336, the mutants 50336△*stnc640* and 50336△*hfq*, complemented mutants 50336△*stnc640*/p*stnc640* and 50336△*hfq*/p*hfq*, and *E.coli* DH5α were grown in Luria-Bertani broth (LB) or on LB agar plates at 37 °C. Strains containing temperature-sensitive plasmids such as pCP20 or pKD46 were grown at 30 °C. Strains harboring antibiotic resistance were cultured in LB containing 100 μg/ml of Ampicillin (Amp) or 34 μg/ml of chloramphenicol (Cm) when appropriate. To determine growth rates, the strains were grown at 37 °C with agitation (180 rpm) in LB broth, and the optical density at 600 nm (OD_600_) was measured every hour. Human colorectal adenocarcinoma epithelial cells (Caco-2) were cultured as described previously [23].

**Table 1:** Bacterial strains and plasmids used in this study

Strains/plasmids	Characteristics	References
Strains		
CMCC(B)50336	*Salmonella enterica* serovar Enteritidis wild-type	NICPBP, China
50336△*stnc640*	*stnc640* deficient mutant	This study
50336△ *stnc640*/*pstnc640*	50336△*stnc640* carrying pBR-*stnc640* (Amp^r^)	This study
50336△*hfq*	*hfq* deficient mutant	[23]
50336△*hfq*/p*hfq*	50336△*hfq* carrying pBR-*hfq* (Amp^r^)	[23]
Plasmids		
pKD3	Cm^r^; Cm cassette teplate	[24]
pKD46	Amp^r^, λRed recombinase expression	[24]
pCP20	Amp^r^, Cm^r^; Flp recombinase expression	[24]
pBR-*stnc640*	pBR322 carrying the full *stnc640* gene (Amp^r^)	This study
pGEM-T Easy	cloning vector, Amp^r^	Takara
pMD19 T-simple	cloning vector, Amp^r^	Takara

### Stability detection of STnc640 in hfq mutants

*S.* Enteritidis WT strain 50336, the mutant 50336△*hfq*, and the complemented mutant 50336△*hfq*/p*hfq* were grown to an OD_600_ of 2.5 and collected by centrifugation. Total RNA was extracted and reverse transcribed to cDNA. The mRNA transcripts of *stnc640* in WT 50336, 50336△*hfq,* and 50336△*hfq*/p*hfq* were detected by real-time quantitative PCR (RT-qPCR) using primers stnc640-F and stnc640-R.

### Prediction of candidate mRNA targets of STnc640

Candidate mRNA targets of STnc640 were predicted using TargetRNA2 [20] (http://old-tempest.wellesley.edu/~btjaden/TargetRNA2/index.html.oldtempest). Using this website, we selected the *Salmonella* Enteritidis strain P125109 genome, input the STnc640 sequence, and then specified 90 nucleotides upstream and 30 nucleotides downstream of the translation start sites of candidate targets. Candidate targets were identified by specifying at least nine consecutive hybridization seeds corresponding to an initial interaction between the sRNA and mRNA with a *p*-value below 0.01.

### Construction of the stnc640 deletion mutant and the complemented strain

The primers used are listed in Table 2. The *stnc640* gene was cloned using PCR primers that flank the *stnc640* gene in *Salmonella* Typhimurium. The construction of *stnc640*-negative mutants of *S.* Enteritidis 50336 was generated by the phage λ-Red-mediated recombination system as described previously [24, 25]. Primers P3 and P4 were used to amplify chloramphenicol resistance-encoding genes to construct the first recombinant strain 50336△*stnc640::cat*. The *stnc640* complete deletion mutant 50336△*stnc640* was confirmed by PCR using primers (P1, P2) and sequencing the PCR product that contained the primers P3 and P4 sequences and lacked of *stnc640* sequences using fluorescence-based chain-termination method with a DNA sequencer ABI 3730XL. The complemented strain was generated by cloning the full-length *stnc640* gene into plasmid pBR322, which was transferred to the *stnc640* mutant. The mutant 50336△*hfq* and complemented mutant 50336△*hfq*/p*hfq* were described previously [23].

**Table 2:** Primers for the PCR and the size of PCR products

Primer	Sequence (5′-3′)	Size (bp)
P1	TGGAAATGGCGGAACATCT	809/416
P2	TAAAGTCAACCCAGGCTCC	
P3	GAAATGTAGTGAGTTTGGTGACGCGATTATCGCAAATATGTAATAACGATGTGTAGGCTGGAGCTGCTTCG	1114
P4	AACCTTTATACTTCCACTATGGCAGATAGGTTTGAGCATATGTCTCCTGACATATGAATATCCTCCTTAG	
P5	CCCAAGCTTCGATTATCGCAAATATGCGA	1591
P6	GCGTCGACTCAGCAGTCTCTATTAAAGCA	
bcfA-F	TGACGCTGCCTGTTCTGTTT	136
bcfA-R	GCAGTCTTCCAGTTTGATGGTG	
fimA-F	GACTGCGATCCGAAAGTGG	91
fimA-R	CAGAGGAGACAGCCAGCAAA	
gyrA-F	GCATGACTTCGTCAGAACCA	278
gyrA-R	GGTCTATCAGTTGCCGGAAG	

### RNA extraction and real-time quantitative PCR

Bacteria were grown to an OD_600_ of 2.5 in LB medium and collected by centrifugation. Total RNA was extracted using TRIzol reagent (Invitrogen, NY, USA). cDNA was synthesized using the PrimeScript RRT reagent kit with gDNA Eraser (Takara Bio, Shiga, Japan). Transcript abundance was quantified using RT-qPCR with SYBR Premix Ex Taq II (Takara) and the primers listed in Table 2 using an ABI7500 instrument (Applied Biosystems, USA). Assays were performed in triplicate, and all data were normalized to the endogenous reference gene *gyrA* using the. 2^-△△CT^ method [26].

### Bacterial adherence and invasion assays

Bacterial adherence and invasion assays were performed as described previously [27]. Bacteria were incubated with a monolayer of 1 × 10^5^ Caco-2 cells at a multiplicity of infection (MOI) of 100 at 37 °C in 96-well tissue culture plates (Corning) for 2 h. Infections were carried out in triplicate. Infected cell monolayers were gently washed three times with PBS to remove loosely adherent bacteria. Cells were lysed with 0.5% Triton X-100 for 30 min. The lysates were serially diluted and plated onto LB agar plates for the enumeration of adherent and invaded bacteria.

### Animal infections

One-day-old chickens (National Chickens Genetic Resources, Yangzhou, China) were randomly divided into one control group and three infection groups (*n* = 20, 10 females and 10 males). *Salmonella* Enteritidis strains 50336, 50336△*stnc640* and 50336△*stnc640* /p*stnc640* were grown to early stationary phase with an OD_600_ of 2.5 in LB medium, harvested by centrifugation, washed, and resuspended to 5 × 10^7^ CFU/mL, 5 × 10^8^ CFU/mL and 5 × 10^9^ CFU/mL gradient suspensions in sterile PBS prior to inoculation into infection group chickens. Three infection groups were separately inoculated with 200 μL 5 × 10^7^ CFU/mL, 5 × 10^8^ CFU/mL or 5 × 10^9^ CFU/mL bacterial suspensions, while the control group received 200 μL PBS by subcutaneous injection. Signs of chickens illness and death were monitored daily. The 50% lethal dose (LD_50_) was calculated 14 days post-infection using the method described previously [23]. Briefly, the numbers of dead and surviving chickens were recorded daily. The summation of cumulative dead and surviving chickens of each dose was taken. The LD_50_ was calculated using the data on percent mortality using the arithmetical method of Reed and Muench [28]. All live chickens were euthanized by pentobarbital after the assays. All procedures complied with institutional animal care guidelines and were approved by the Animal Care and Ethics Committee of the Yangzhou University (Approval ID: YZUDWSY2017–0026).

### Statistical analysis

Data were analyzed using Student’s *t* test for independent samples. Differences were considered significant if *P* ≤ 0.05.

## Data Availability

The datasets used and analysed during the current study are available from the corresponding author on reasonable request.

## Abbreviations

Amp: Ampicillin
Caco-2: Human colorectal adenocarcinoma epithelial cells
Cm: Chloramphenicol
IGRs: Intergenic regions
LB: Luria-Bertani broth
LD_50_: 50% lethal dose
OD_600_: Optical density at 600 nm
RT-qPCR: Real-time quantitative PCR
sRNAs: Small non-coding RNAs
WT: Wild type
